# Early Postoperative C-Reactive Protein Trajectories After Thoracic Surgery: A Retrospective Cohort Study

**DOI:** 10.3390/biomedicines13102532

**Published:** 2025-10-17

**Authors:** Ilker Kolbas, Berivan Karatekin, Esra Ergun Alış, Irfan Cicin

**Affiliations:** 1 Department of Thoracic Surgery, Istanbul Aydin University Florya Medical Park Hospital, Istanbul 34295, Turkey; 2 Department of Pulmonology, Istanbul Aydin University Florya Medical Park Hospital, Istanbul 34295, Turkey; berivankaratekin@gmail.com; 3 Department of Infectious Disease, Istanbul Aydin University Florya Medical Park Hospital, Istanbul 34295, Turkey; ergunesra03@yahoo.com; 4 Department of Medical Oncology, Istinye University, Istanbul 34000, Turkey; irfancicin@gmail.com

**Keywords:** C reactive protein, thoracic surgery, VATS, thoracotomy, postoperative infection, biomarker kinetics

## Abstract

**Background:** Distinguishing expected postoperative inflammation from early infection remains challenging after thoracic surgery; serial C-reactive protein (CRP) is widely used to aid this differentiation. **Methods:** We conducted a single-centre retrospective cohort study of adults undergoing thoracic surgery (1 January 2022–31 December 2024). CRP was measured preoperatively and on postoperative days (POD) 1–5; trajectories were compared by surgical approach and extent of resection using repeated-measures ANOVA with Greenhouse–Geisser correction (α = 0.05). **Results:** Among 144 patients (VATS *n* = 79; open thoracotomy *n* = 65; extent: segmentectomy *n* = 25, lobectomy *n* = 96, bilobectomy *n* = 9, pneumonectomy *n* = 14), overall CRP rose from 26.6 ± 45.0 mg/L preoperatively to a POD2 peak of 200.9 ± 72.7 mg/L, then declined to 118.1 ± 70.7 mg/L by POD5. Thoracotomy showed higher peaks than VATS (POD2 216.1 ± 76.0 vs. 152.3 ± 29.9 mg/L; POD3 206.7 ± 88.7 vs. 159.8 ± 72.4 mg/L), but time × approach was not statistically significant (F = 1.042, *p* = 0.381; partial η^2^ = 0.115). The extent analysis showed the highest peaks with pneumonectomy (POD2 273.7 ± 46.3 mg/L) compared with bilobectomy (155.7 ± 11.0 mg/L) and lobectomy (VATS 132.1 ± 3.7, open 196.8 ± 85.3 mg/L); time × extent was not significant (F = 1.136, *p* = 0.384; partial η^2^ = 0.299). The overall effect of time did not reach significance (F = 1.127, *p* = 0.352; partial η^2^ = 0.124), reflecting variability. Patients with clinically diagnosed infections exhibited more prolonged CRP elevation, often >100 mg/L beyond POD4, whereas uncomplicated cases declined after the POD2 peak; these trends did not achieve statistical significance in this cohort. **Conclusions:** Early postoperative CRP in thoracic surgery typically peaks at 48–72 h and then falls. Higher peaks with open surgery and more extensive resection were observed but not statistically confirmed; persistence > 100 mg/L after POD3–4 may flag complications. Prospective studies are needed to validate thresholds and refine CRP-based surveillance pathways.

## 1. Introduction

Postoperative infections remain a major concern in surgical patients, contributing significantly to morbidity and healthcare costs. Globally, an estimated 6.5% to 18% of patients will develop a postoperative infection within the first 30 days after surgery [1,2]. Thoracic surgery patients, in particular, are at risk for pulmonary infections (such as pneumonia or empyema) and surgical site infections due to the invasive nature of lung resections and the frequent need for extensive lymph node dissection. Early identification of such infections is critical for timely intervention yet also challenging because the early postoperative period is characterized by systemic inflammatory responses related to the surgical trauma itself. Clinicians must distinguish an expected postoperative inflammatory reaction from the onset of sepsis, so as to treat genuine infections promptly while avoiding unnecessary antibiotics in sterile inflammatory conditions [3,4,5,6].

CRP is one of the most commonly used biomarkers to monitor inflammation and possible infection after surgery. CRP is an acute phase protein synthesized by the liver in response to pro-inflammatory cytokines (primarily interleukin-6) released during tissue injury, infection, or other inflammatory stimuli [7,8,9]. In healthy individuals, baseline CRP levels are very low (typically <3 mg/L, up to ~10 mg/L at most) [10]. Nearly all surgical procedures provoke a rise in CRP due to tissue damage; this is a normal part of the healing process. The typical postoperative CRP kinetic profile consists of an initial increase starting within hours after surgery (CRP production begins ~6–8 h after insult), with peak concentrations reached around the second to third postoperative day [7,8,9]. Thereafter, if recovery is uncomplicated, CRP levels begin to decline steadily, often returning toward baseline by about one week post-surgery [11]. This predictable pattern has been observed across various surgical disciplines. For example, in a large cohort study of surgical patients, CRP peaked on day 3 on average and then gradually fell, reflecting the resolution of surgical inflammation in uncomplicated cases [1].

The challenge is that early postoperative CRP elevations are non-specific—a high CRP in the first 2–3 days after surgery could be entirely due to the surgical trauma rather than infection. In the immediate post-surgical period, virtually all patients have CRP values well above normal; indeed, peak CRP levels around 100–200 mg/L by postoperative day 2 are common even in patients without any complications [12]. For instance, one study in colorectal surgery reported mean CRP peaks of ~140 mg/L on day 2 in patients with uneventful recovery [12]. Thus, using an absolute CRP cutoff in the first 48 h may be misleading. However, differences begin to emerge after the initial peak period: patients who develop infections or other inflammatory complications often exhibit higher peaks or a failure of CRP to decline in the later postoperative days compared to those without complications [13]. CRP kinetics beyond post-op day 3 can therefore provide important clues. A persistently elevated CRP or a second rise in CRP levels after the expected peak has been identified as a warning sign of postoperative infection in several studies. Santonocito et al. (2014) prospectively observed that in patients who remained infection-free after major surgery, CRP typically peaked by day 3 and then significantly decreased, whereas in those who developed sepsis or severe infection, CRP levels were not only higher from the outset but remained elevated past day 4 [13]. Notably, CRP levels > 100 mg/L after postoperative day 4 had a high association with the presence of an infection or sepsis in their cohort. This aligns with clinical intuition that by 4–5 days after surgery, CRP should start trending down; if it does not, one should suspect an ongoing inflammatory complication. Babic et al. (2020) demonstrated this in patients undergoing esophagectomy: those who had open operations showed an earlier and more pronounced CRP rise than those who had minimally invasive procedures [14]. In that study, a CRP value exceeding 200 mg/L on postoperative day 2 was an independent early predictor of major complications (including pulmonary infections and anastomotic leakage) [15]. Similarly, other researchers have proposed CRP thresholds on day 3 or day 4 (ranging roughly 150–220 mg/L) that can alert clinicians to a high risk of infectious complications, such as pneumonia or surgical site infection, following lung cancer surgery [16]. On the other hand, a normalizing or low CRP in the late postoperative course has high negative predictive value for complications [15]—in other words, if CRP drops below certain cut-offs by day 4–5, the likelihood of an occult infection is low. This makes serial CRP measurements a potentially useful tool in guiding postoperative management: patients with reassuring CRP trends might be considered for early discharge, while those with aberrant trends receive closer monitoring [17].

Despite these insights, the actual utility of CRP monitoring in thoracic surgery is not fully established, and some studies have yielded mixed results regarding its discriminatory power [1,18,19,20]. The inflammatory response can vary greatly between individuals, and factors such as preoperative inflammation (due to the tumor or chronic lung disease) can elevate baseline CRP and cloud postoperative interpretation [15]. Moreover, most published data focus on specific operations like esophagectomy or colorectal surgery; there is a relative paucity of data focusing on CRP kinetics after pulmonary resection (lung surgery) and how these relate to routine postoperative recovery versus complications. We identified this gap in the literature and the need for a detailed evaluation of CRP dynamics in general thoracic surgical patients.

Given this background, we designed the present study to comprehensively analyze postoperative CRP trends in a cohort of thoracic surgery patients and to determine whether CRP patterns differ by surgical approach or correlate with the occurrence of infections. We hypothesized that the magnitude of CRP elevation would be primarily driven by the extent of surgical tissue trauma (with higher peaks in open and extensive surgeries) and not inherently indicative of infection unless the elevated levels persist beyond the immediate postoperative period. By examining CRP kinetics alongside clinical outcomes, our goal was to assess the role of CRP in early detection of postoperative infectious complications in thoracic surgery. This study aims to help clarify how clinicians can interpret CRP changes in the days following lung surgery—distinguishing normal postoperative inflammatory responses from unusual patterns that might warrant further diagnostic work-up for infection.

## 2. Materials and Methods

### 2.1. Study Design and Setting

This study was a retrospective observational analysis of postoperative CRP kinetics in thoracic surgery patients. It was conducted at Istanbul Aydin University Medical Park Florya Hospital, a tertiary care hospital, and received approval from the hospital’s Institutional Review Board (approval no. 142/2025). The requirement for informed consent was waived due to the retrospective design, in accordance with ethical guidelines. We adhered to the Strengthening the Reporting of Observational Studies in Epidemiology (STROBE) guidelines for reporting cohort studies [21].

### 2.2. Patient Selection

We included all adult patients (age ≥ 18 years) who underwent a major thoracic surgical procedure between 1 January 2022 and 31 December 2024 at our institution. Thoracic surgery was defined as any operation performed by the thoracic surgery service, predominantly consisting of pulmonary resections for lung tumors (e.g., wedge resection, segmentectomy, lobectomy, bilobectomy, pneumonectomy), as well as a smaller number of other procedures (such as decortication or exploratory thoracotomy if present). Patients who underwent minimally invasive surgery (VATS or robotic-assisted thoracoscopy) and those who had open thoracotomy were both included. We excluded cases if key data on CRP levels were missing (e.g., if a patient was transferred or discharged before any postoperative labs were obtained) or if the patient had a documented active infection at the time of surgery (since that would confound postoperative CRP interpretation). Emergency surgeries (if any occurred in the thoracic unit during the study period) were also excluded to maintain a relatively homogeneous elective surgical cohort. After applying these criteria, 144 patients met inclusion for analysis.

We achieved a largely homogeneous sample focused on lung resections. Only 2% of included cases (3 patients) were non–lung resection procedures (isolated pleural decortication). We confirmed that their inclusion did not materially change the CRP trend results; thus, we retained them to preserve sample size.

Thoracic surgery was defined as any operation performed by our thoracic service, predominantly consisting of pulmonary resections for lung tumors. Specifically, approximately 75% of patients underwent resection for a primary lung carcinoma (NSCLC), 15% underwent metastasectomy (wedge or lobar resection of metastatic lesions), and the remainder had other thoracic procedures (benign tumor resection or empyema decortication). We did not include any cases of chemical pleurodesis in this series. Surgical interventions ranged from minor wedge resections and segmentectomies to lobectomies, bilobectomies, and pneumonectomies. A small number of complex cases had additional procedures (one patient with pleural empyema underwent open decortication).

### 2.3. Data Collection

This study utilized retrospective chart review and electronic medical records to obtain necessary data. A standardized data extraction form was used to record patient characteristics (age, sex, and relevant comorbidities), surgical details, laboratory results, and outcomes. Surgical details recorded included the surgical approach (categorized as either minimally invasive—primarily VATS—or open thoracotomy) and the extent of resection (categorized as minor resection [wedge or segmentectomy], lobectomy, or more extensive resection [bi-lobectomy or pneumonectomy]). We also noted if a mediastinal lymph node dissection or staging mediastinoscopy was performed, as this could contribute to inflammatory response. Histopathological diagnosis and tumor stage (for oncology cases) were recorded, but these were analyzed mainly for completeness and secondary correlations.

For each patient, CRP levels were recorded at the following time points: the preoperative baseline (usually the last value drawn the day before surgery or on the morning of surgery, labeled postoperative day 0), and daily on postoperative days 1 through 5. All CRP measurements were those routinely ordered by the surgical team as part of postoperative care; in our institution it is typical to measure CRP (and complete blood count) daily for at least the first few days after major surgery. Serum CRP was measured in the hospital laboratory using an immunoturbidimetric assay (normal reference range < 5 mg/L). We also collected white blood cell (WBC) counts at the same postoperative intervals to compare with CRP trends, as WBC is another common inflammation marker. Additionally, serum albumin levels on postoperative days 0–5 were recorded, given that hypoalbuminemia might indicate systemic inflammation or poor nutritional status.

Comorbidity data (chronic obstructive pulmonary disease, diabetes mellitus, autoimmune/rheumatologic conditions) were not systematically recorded in structured fields during the study period and could not be extracted with adequate reliability from free-text clinical notes or scanned documents; therefore, comorbidities were not analyzed in this study. Detailed postoperative course variables—such as length of stay, day of clinical diagnosis and antibiotic initiation, non-infectious complications, and 30-day outcomes—were not systematically recorded in structured form during the study period and could not be reliably abstracted from free-text or scanned sources; accordingly, these variables were not analyzed. Where documented by the treating team, postoperative infection was captured as a binary outcome (present/absent), but granular timing data were unavailable.

### 2.4. Outcome Measures and Definitions

The primary outcomes of interest were the trends in CRP over time after surgery and how these trends differed by (a) surgical approach and extent and (b) occurrence of postoperative infection. We defined postoperative infection as any clinically significant infection occurring during the hospital stay or within 30 days of surgery that required treatment. This included pulmonary infections (such as pneumonia or bronchopulmonary infections confirmed by clinical exam and imaging, with or without positive cultures), pleural space infections (empyema), surgical site infections (incisional wound infection or deeper thoracic infection), or systemic infections like sepsis/bacteremia originating in the postoperative period. For the purpose of this study, we relied on documentation in the charts: if the treating physicians diagnosed an infection and initiated antimicrobial therapy (antibiotics) or performed an intervention (e.g., abscess drainage), we counted that as a postoperative infection [1]. The date of infection onset was noted, as well as the type of infection. We also recorded antibiotic use—specifically, whether broad-spectrum antibiotics were started and on which postoperative day—as a proxy indicator for suspected infection. Other postoperative complications (e.g., atrial fibrillation, prolonged air leak, etc.) were noted but were not the primary focus of this analysis. Mortality was tracked up to 30 days post-surgery; any in-hospital death was recorded with cause.

### 2.5. Statistical Analysis

All data were analyzed using IBM SPSS Statistics software (IBM Corp., Armonk, NY, USA; Version 28.0). Continuous variables were summarized as mean ± standard deviation or median (interquartile range) as appropriate based on distribution, and categorical variables as frequencies (percentages). We first performed descriptive analyses of CRP values at each time point overall and stratified by surgical groups. For inferential analysis, we employed a repeated-measures ANOVA to assess changes in CRP over time (from preoperative baseline through postoperative day 5) and to test for differences in the CRP trajectory between groups. In the ANOVA model, “time” (POD0, POD1, POD5) was the within-subject factor, and we included between-subject factors for surgical approach (VATS vs. open) and extent of resection (segmentectomy/wedge, lobectomy, vs. bi-lobectomy/pneumonectomy), as well as the presence or absence of postoperative infection. The repeated-measures analysis allowed us to evaluate interaction terms (time × group) to see if CRP trends over days differed significantly by group.

This retrospective study was not designed or powered to perform post hoc subgroup analyses based on CRP persistence after POD2. To avoid time-dependent selection bias and multiplicity, we did not conduct unplanned analyses stratifying patients by “persistent CRP” beyond POD2; instead, we focused on a priori outcomes (overall early CRP kinetics and between-group comparisons). Mauchly’s test was used to check the sphericity assumption; if sphericity was violated, we applied Greenhouse–Geisser corrections for the F-tests. For simplicity of interpretation, we also conducted two-sample comparisons at specific time points: for instance, we used independent-samples t-tests (or nonparametric Mann–Whitney U tests if distributions were skewed) to compare mean CRP on each postoperative day between VATS vs. open surgery, and between patients with vs. without infection. Categorical variables (e.g., infection rates between VATS and open groups) were compared with chi-square or Fisher’s exact tests. A two-tailed *p*-value < 0.05 was considered statistically significant for all analyses.

## 3. Results

**Figure 1:**
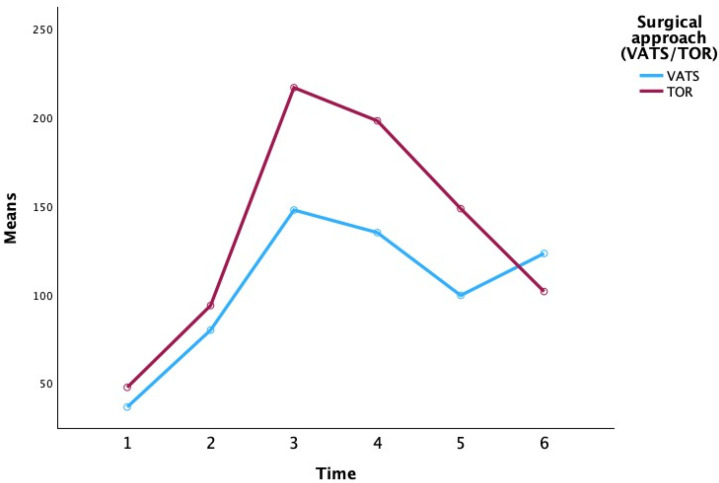
**Comparison of CRP levels over time between surgical approach groups (VATS vs. open thoracotomy).** Each line represents the mean CRP for the group at each time point (P0: preoperative, P1–P5: postoperative days 1–5). Both groups exhibit a post-surgical rise in CRP, peaking around postoperative day 2 (P2) and gradually declining thereafter. The open thoracotomy group shows a higher peak CRP (mean ~216 mg/L at P2) compared to the VATS group (mean ~152 mg/L), although this difference was not statistically significant. By day 5 (P5), CRP levels in the two groups have converged, with both approaching baseline levels.

The study cohort had a mean age of 58.3 ± 12.4 years, with 60% male and 40% female patients. The median BMI was 26.1 (IQR 24–29), and 65% of patients had a history of smoking (40% current or recent smokers). Detailed demographics are presented in Table 1. Briefly, the VATS and open surgery groups were similar in age and BMI (no significant difference, *p* > 0.10), and the proportion of males was slightly higher in the open thoracotomy group (67% vs. 55%). We conducted a repeated-measures analysis of variance (ANOVA) to examine changes in CRP levels over time and to assess whether these changes differed by surgical approach (video-assisted thoracoscopic surgery, VATS, vs. open thoracotomy, TOR) (Figure 1) and extent of resection (segmentectomy, lobectomy, pneumonectomy, bilobectomy).

**Figure 2:**
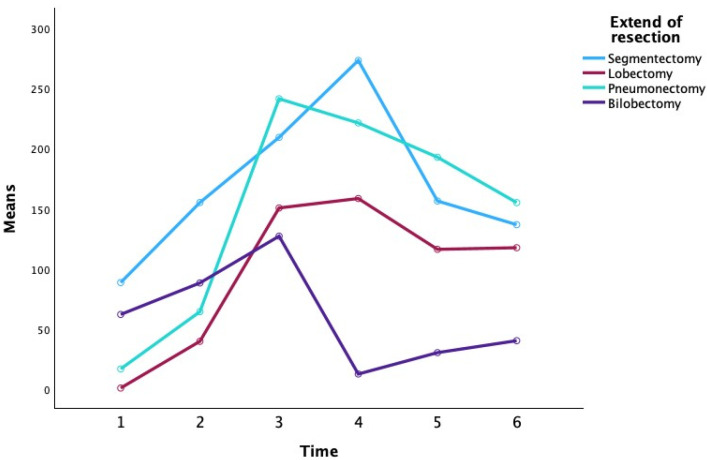
**Comparison of CRP levels over time among extent of resection groups.** Mean CRP at each time point is plotted for patients undergoing segmentectomy, lobectomy, pneumonectomy, or bilobectomy. All groups demonstrate a similar temporal pattern: CRP rises after surgery to a peak around day 2–3 (P2–P3) and then declines toward day 5. The pneumonectomy group tended to have the highest CRP values at the peak (~250+ mg/L at P2–P3), whereas the lesser resection groups (segmentectomy, lobectomy, bilobectomy) showed lower peaks (approximately 140–190 mg/L). By postoperative day 5, CRP levels had decreased substantially in all groups, and differences between groups had diminished. These trends were not statistically significant, indicating no reliable difference in CRP kinetics by extent of resection.

CRP was measured at six time points (preoperatively on day 0, and postoperatively through day 5). Additional patient factors (age, sex, use of mediastinoscopy, tumor location, side, histological subtype, and tumor staging T/N) were included in the model as between-subject factors or covariates. All assumptions for repeated-measures ANOVA were checked. In particular, Mauchly’s test was used to verify the sphericity assumption for the within-subject factor (time). Where sphericity was violated, Greenhouse–Geisser corrections were applied to the degrees of freedom. A significance level of α = 0.05 (two-tailed) was used for all statistical tests. Table 2 presents the descriptive statistics for CRP at each time point, stratified by surgical approach and extent of resection (Figure 2).

At the preoperative baseline (P0), mean CRP levels were relatively low in most groups (e.g., 3.25 ± 1.63 mg/L for VATS lobectomy and 13.35 ± 13.52 mg/L for open lobectomy), with the exception of the VATS segmentectomy subgroup which showed a higher mean (80.45 ± 107.69 mg/L), likely reflecting an outlier or preoperative inflammatory condition. By the first postoperative day (P1), CRP had risen markedly in all groups (overall mean 71.47 ± 47.04 mg/L), consistent with an acute post-surgical inflammatory response. CRP continued to increase to a peak around the second postoperative day (P2) for most groups. Notably, patients who underwent TOR showed a higher peak CRP on day 2 (mean 216.12 ± 76.04 mg/L) compared to those who underwent VATS (mean 152.32 ± 29.86 mg/L). Among the extent-of-resection groups, pneumonectomy was associated with the highest CRP values at the peak (mean 251.55 ± 68.32 mg/L at P2), whereas lesser resections like lobectomy and segmentectomy had lower peak means (approximately 184–187 mg/L at P2–P3). By postoperative day 3 (P3), CRP levels plateaued or began to decline in most groups. In the subsequent days (P4 and P5), CRP concentrations declined substantially. By day 5, the differences between surgical approaches had narrowed (mean CRP 132.72 ± 86.12 mg/L for VATS vs. 113.49 ± 67.73 mg/L for TOR), and CRP levels in all extent groups had returned closer to baseline (e.g., segmentectomy 82.87 ± 50.17 mg/L, lobectomy 112.77 ± 80.99 mg/L, pneumonectomy 158.00 ± 60.04 mg/L, bilobectomy 77.55 ± 30.90 mg/L at P5). Overall, Table 2 indicates that CRP followed a typical postoperative kinetic pattern, peaking around 48 h after surgery and declining thereafter, with some suggestion that more invasive surgery (open approach or greater resection) produced higher CRP elevations, although these are descriptive trends without inferential statistics in this table.

Inferential statistics from the repeated-measures ANOVA are summarized in Table 2, Table 3 and Table 4. The multivariate tests for the within-subject factor (time) and its interactions (Table 3) revealed no statistically significant effect of time on CRP overall, and no significant time–group interactions. In particular, there was no significant overall change in CRP across the six time points when considered for all patients (Pillai’s Trace = 0.413, F = 0.562, *p* = 0.730; Table 3). Likewise, the pattern of CRP over time did not differ significantly between the surgical approach groups (time × surgical approach interaction: Pillai’s Trace = 0.524, F = 0.882, *p* = 0.564) or among the extent of resection groups (time × extent interaction: Pillai’s Trace = 0.885, F = 0.502, *p* = 0.908). None of the other patient factors showed a significant interaction with time either: for example, there was no significant time × age interaction (Pillai’s Trace = 0.353, *p* = 0.806) and no time × sex interaction (Pillai’s Trace = 0.484, *p* = 0.627), indicating that the trajectory of CRP over time was consistent regardless of patient age or gender. Similarly, the presence of mediastinoscopy, tumor position or side, histology, or tumor stage (T/N) did not significantly modulate the CRP time-course (all interactions with time non-significant, *p* > 0.05 in Table 3). Although some of these factors showed moderate effect size estimates (e.g., Pillai’s Trace for time × N stage = 0.651, suggesting a substantial proportion of variance), the associated *p*-values were all well above 0.05, likely due to high variability and small subgroup sample sizes, and thus did not reach statistical significance.

The univariate (within-subject) ANOVA results, reported in Table 4, were consistent with the multivariate findings. Using a repeated-measures ANOVA with sphericity corrections, we found no significant main effect of time on CRP levels. The sphericity assumption was examined for the factor of time (6 levels); Mauchly’s test indicated violation of sphericity, so the Greenhouse–Geisser adjusted results were considered. The Greenhouse–Geisser adjusted F-ratio for the effect of time was *F*(2.251, *ε*) = 1.127, *p* = 0.352 (partial η^2^ = 0.124), confirming that CRP did not change significantly over time when averaged across all groups. Similarly, the time × surgical approach interaction was not significant (e.g., using the Greenhouse–Geisser correction, *F* ≈ 1.042, *p* = 0.381; Table 4). The interaction between time and extent of resection also failed to reach significance (*F* ≈ 1.136, *p* = 0.359 for sphericity assumed; Greenhouse–Geisser *p* = 0.384). In fact, none of the interactions between time and the other factors (age, gender, mediastinoscopy, tumor position, side, histology, T stage, or N stage) showed a statistically significant effect on the CRP trajectory (all *p* > 0.25 after appropriate corrections; see Table 4). These results indicate that, despite observed numeric differences in mean CRP between groups (as seen in Table 2), there was substantial variability and overlap such that no significant group differences in the pattern of CRP change over time could be confirmed.

Finally, Table 5 provides a more detailed look at specific pairwise comparisons between time points (within-subject contrasts) and how these comparisons differ by group. Consistent with the above results, no significant differences were found between any two consecutive time points in overall CRP levels. For example, the increase from P0 to P1 was not statistically significant (*F* = 0.246, *p* = 0.633), nor was the rise from P1 to P2 (*F* = 2.319, *p* = 0.166), and none of the subsequent pairwise time-point differences reached significance (all *p* > 0.25; Table 5). The lack of significant contrasts confirms that the mean postoperative CRP peaked and then declined without any single time interval showing a significant jump or drop across the sample as a whole. In addition, there were no significant time-point differences between the surgical approach groups (time × approach contrasts). For instance, the change in CRP from P1 to P2 did not differ significantly between VATS vs. open surgery (*F* = 1.532, *p* = 0.251 for the interaction contrast), and similarly for all other consecutive intervals (all *p* > 0.05). We note that the largest approach-related difference in CRP change was observed between P4 and P5, where CRP in the open thoracotomy group continued to decline while the VATS group showed a slight increase; however, this difference was only marginal (*F* = 4.998, *p* = 0.056) and did not reach the significance threshold. No significant differences in CRP change were detected among the extent of resection groups for any pair of time points (all time × extent contrast *p* > 0.24). For example, the degree of CRP rise from P1 to P2 did not significantly depend on whether patients had a segmentectomy, lobectomy, pneumonectomy, or bilobectomy (*p* = 0.249 for the interaction; Table 5). Likewise, none of the contrasts for time × age, time × sex, or other factors were significant, indicating that the pattern of increase and decrease in CRP was statistically parallel across all subgroups. In summary, the inferential tests demonstrate that the postoperative CRP kinetic profile did not differ significantly by surgical approach or extent of resection, despite numerical trends, and that the CRP changes over time were not significantly influenced by patient demographic or tumor-related factors in this sample.

Across the cohort, CRP rose after surgery, typically peaking around POD2 and subsequently declining in uncomplicated recoveries. Patients with clinically diagnosed infections demonstrated a qualitatively more prolonged inflammatory course, consistent with a slower CRP decline; however, formal between-group differences did not reach statistical significance in our a priori analyses. Detailed postoperative clinical course metrics (length of stay, timing of infection diagnosis and antibiotic initiation, non-infectious complications) were unavailable in structured form and were therefore not analyzed; results are focused on serial CRP kinetics across POD0–POD5 (and infection status where recorded).

## 4. Discussion

Our retrospective cohort study examined CRP kinetics after thoracic surgery and found characteristic patterns that mirror the acute postoperative inflammatory response reported in the literature. In our cohort, CRP levels rose rapidly to a peak around postoperative day 2, then gradually declined in patients with an uncomplicated recovery. This timing corresponds to the well-described acute phase surge from surgical trauma: prior studies across various surgical disciplines have shown that CRP typically reaches its maximum approximately 48 h after surgery and then decreases promptly as the inflammatory stimulus resolves [22,23]. Our findings are consistent with this normal trajectory, as patients without complications demonstrated a steady fall in CRP after day 2. In contrast, those who developed postoperative infections tended to exhibit persistently elevated CRP levels beyond postoperative day 4 (often remaining > 100 mg/L). Although the CRP differences between infected and uncomplicated cases in our sample did not reach statistical significance, the observed trend—namely, a failure of CRP to normalize in the late postoperative period—is clinically suggestive of an underlying infectious or inflammatory complication. This interpretation aligns with the concept that a secondary rise or plateau in CRP after the initial postoperative peak can signal occult infection or surgical-site complication [22,24]. In other words, while an early CRP peak is expected from surgical injury alone, an abnormally prolonged or rebound CRP elevation may indicate that the patient’s inflammatory response is being sustained by infection or another pathology. Even though our study did not show a statistically significant separation in CRP values, the qualitative pattern of persistent CRP elevation in complicated cases underscores a potential warning sign that merits clinical attention, in line with prior observations [22,25].

When placing our findings in context, we note strong agreement with existing research on postoperative CRP trends. Numerous studies have documented that CRP rises quickly after major surgery and then declines in the absence of complications. For example, Kallio et al. reported that CRP peaked on postoperative day 2 after orthopedic fracture surgery, following which it dropped steadily in uncomplicated cases [26]. This normal kinetic profile has been observed across orthopedic, cardiac, and abdominal procedures, with peak CRP values typically occurring on day 2–3 and returning toward baseline by around postoperative days 7–14 in uneventful recoveries [22]. Our thoracic surgery cohort exhibited a similar early peak and subsequent decline, reinforcing that thoracic procedures elicit an acute-phase CRP response on a timetable comparable to other major surgeries. Moreover, the divergence in CRP trajectories between uncomplicated and complicated cases that we observed has also been reported in prior studies. In a head-and-neck reconstructive surgery series, for instance, patients with a normal recovery showed CRP peaking around day 2 and then falling, whereas those with postoperative complications had CRP levels that rose again or failed to drop after day 3, a difference that was statistically significant in that study (with CRP dynamics yielding ~83% sensitivity and 78% specificity for early complication detection) [27,28,29]. Similarly, a meta-analysis by Adamina et al. (2015) focusing on abdominal surgeries found that CRP measurements on postoperative day 4 have high prognostic value—in particular, a normalizing CRP by day 4 had a high negative predictive value (~84%) for ruling out infectious complications [30,31,32]. This means that when CRP is low or trending downward by the fourth day, the likelihood of a significant infection is low, whereas persistently high CRP at that point is cause for concern. In our study, CRP > 100 mg/L beyond day 4 was frequently observed in patients who ultimately were diagnosed with infections, which is congruent with these reports (even though our sample size limited the statistical power). Indeed, various investigations in surgical patients have proposed CRP threshold values in the range of ~100–150 mg/L in the late postoperative period as “red flags” for possible complications [33]. For example, a recent analysis of abdominal wall surgery patients determined that a CRP level around 100 mg/L or higher in the first week optimally discriminated those with complications (with ~80% sensitivity and 95% specificity) [34]. Consistently, the authors recommended that patients with CRP > 100 mg/L in the early postoperative course be considered at high risk for an occult complication and undergo thorough evaluation (e.g., imaging) [33]. Our data support this general threshold, as none of the uncomplicated cases maintained CRP in the >100 mg/L range after day 4, whereas several infected cases did. Thus, while not conclusive on its own, our study’s CRP kinetics align with the broader literature underscoring that “CRP should go down after day 3–4” in normal recovery and that continued elevation is a warning sign of potential infection.

It is important to note, however, that findings across studies are not entirely uniform, and CRP is not a perfect stand-alone discriminator of postoperative infection. Our results did not reach statistical significance for CRP differences, highlighting the challenges and variability in using a single inflammatory marker to detect complications. This could be partly due to sample size limitations or heterogeneity in our cohort. In some contexts, other investigators have likewise found CRP to have limitations. For instance, in thoracic surgical patients with an existing infection (pleural empyema requiring surgery), one study reported that CRP levels remained elevated in the first week after surgery and declined only slowly, with no significant difference between patients who recovered well and those who required further intervention. In that setting—essentially an active infection being surgically managed—the CRP was uniformly high and thus less informative for predicting outcomes. Another nuance is that CRP can be elevated for many reasons besides infection, including the normal tissue injury of surgery, inflammatory conditions, or minor complications that do not progress. This inherent lack of specificity means that false-positive elevations can occur, potentially triggering unnecessary investigations if CRP is interpreted in isolation. Conversely, false negatives are also possible—for example, a well-localized infection or abscess might not cause a dramatic systemic CRP response in certain patients. The optimal use of CRP thus involves interpreting trends over time and considering the entire clinical picture, rather than relying on any single postoperative CRP reading. Encouragingly, most evidence suggests that the trend (direction) of CRP change is more informative than absolute values—patients whose CRP fails to decrease after the typical peak are more likely to harbor complications [22,35]. Our study reinforces this idea qualitatively, even though we did not reach significance, and it resonates with the principle reported by others that “an abnormal CRP trend” (such as a second rise or persistent plateau) is an early indicator of trouble [22,35,36]. On the other hand, we also acknowledge recent prospective data that question how much early postoperative CRP alone can really single out impending infections in thoracic surgery. In a two-center study of lung cancer surgery patients, investigators found that CRP (and IL-6) levels rose in all patients postoperatively, including those who never developed any infection, meaning the markers’ additional predictive value was limited in the very early period. Those authors concluded that better early-warning biomarkers are needed—a reminder that while CRP is a useful tool, it is not infallible. Taken together, the literature suggests that CRP kinetics are a helpful adjunct for postoperative monitoring, but they must be interpreted with caution and in context, and ideally in combination with other clinical findings or tests.

Clinical implications: The patterns observed in CRP levels after thoracic surgery have direct practical implications for postoperative care. First and foremost, serial CRP measurements can serve as a cheap, readily available adjunct to clinical monitoring in the days following major surgery. CRP testing is inexpensive and widely accessible (on the order of only a few dollars per test) [22,37], making it feasible to incorporate into routine postoperative bloodwork. Our findings support using CRP trends as an additional “vital sign” during recovery—much like temperature or white cell count—to flag patients who might be developing a complication. In practice, a CRP that peaks around day 2 and then substantially decreases by day 4–5 is a reassuring sign, concordant with uncomplicated recovery [22,38]. This scenario could give the clinician greater confidence in moving forward with postoperative milestones (e.g., mobilization, discharge planning), especially if the patient’s clinical exam is also benign. In fact, prior work in abdominal surgery suggests that patients with sufficiently low CRP levels by postoperative day 3–4 have a very low probability of harboring serious infection, and thus early discharge may be considered safe in such cases [39]. On the other hand, persistently high or rising CRP in the late postoperative period should prompt heightened vigilance and evaluation. In a thoracic surgery context, this might entail a careful search for sources of infection—for example, chest imaging to look for pneumonia or anastomotic leak (in esophageal cases), examination of surgical incisions for occult abscess or empyema, and consultation with the broader care team to correlate any other signs (fever, respiratory symptoms, etc.). Importantly, CRP is not meant to replace clinical judgment or diagnostic imaging, but rather to complement them. An abnormal CRP trend serves as an early warning signal that “something might be wrong” even before overt symptoms occur [22]. Our data suggest that if CRP remains > 100 mg/L beyond about postoperative day 4, the index of suspicion for infection should rise. In such situations, proactive steps—such as obtaining a CT scan to rule out a hidden abscess or initiating broad-spectrum antibiotics while awaiting confirmatory tests—may be justified in order to intervene early [33]. Conversely, if CRP is trending downward appropriately, one might avoid unnecessary interventions in a patient who is otherwise doing well. In summary, monitoring CRP kinetics can aid in tailoring postoperative management: a normalizing CRP can reinforce confidence in recovery and possibly shorten hospital stay, whereas an aberrant CRP pattern should alert clinicians to dig deeper for potential complications. Ultimately, using CRP in this way may improve timely detection of problems and thus improve patient outcomes, as long as it is applied thoughtfully alongside clinical assessment.

Building on these findings, further studies are warranted to enhance the utility of CRP kinetics in postoperative care, especially in thoracic surgery. One priority is to conduct prospective studies to validate specific CRP thresholds and time-points as predictors of complications. Our study and many others have been retrospective; a prospective design would allow standardized CRP monitoring and timely correlation with detected infections, helping to establish evidence-based cutoff values (for example, confirming whether CRP > 100 mg/L on postoperative day 4–5 truly predicts thoracic complications with high sensitivity and acceptable false-positive rates). It may also be valuable to stratify such analysis by procedure type (e.g., lung resection vs. esophagectomy vs. thoracotomy for infection) since the inflammatory magnitude and complication profile can differ between these groups. Another avenue for research is exploring composite or multi-marker approaches. While CRP alone is useful, combining it with other biomarkers might improve diagnostic accuracy. Candidates include procalcitonin (PCT), interleukin-6 (IL-6), and other cytokines or acute-phase reactants that rise in infection. Early investigations have yielded mixed results—for instance, some studies in abdominal surgery found that PCT offered no clear advantage over serial CRP for early leak detection [40,41,42,43], and recent work in thoracic surgery noted that adding IL-6 provided only limited predictive boost beyond CRP. Nonetheless, it remains plausible that certain markers (or combinations thereof) could detect specific complications sooner or more specifically. Future research could examine risk-prediction models that integrate CRP trends with other variables (such as vital signs, clinical risk scores, or novel biomarkers) to produce a more robust early warning system for postoperative infection. Additionally, investigations into the kinetic patterns (e.g., rate of CRP decline, or a secondary “bounce” in CRP) and how these might differ between types of complications could refine how we interpret the data; for example, an anastomotic leak might cause a different CRP trajectory than a superficial wound infection. Finally, given the immunologic aspects of cancer and major surgery, research into how patient-specific factors (like baseline inflammatory status, use of steroids or immunosuppressants, and tumor-related inflammation) modulate CRP responses would be valuable. This could help adjust CRP threshold interpretation for individual patient contexts. In summary, future studies—ideally large, multicenter and prospective—should seek to confirm the optimal CRP cutoffs, timing, and integration with other tools to maximize the sensitivity and specificity for early complication detection in thoracic surgery. Such efforts could ultimately lead to improved postoperative protocols, whereby CRP kinetics are incorporated into validated algorithms for risk stratification and decision-making.

Despite the lack of statistically significant differences, the patterns observed in our data are clinically and biologically plausible. Open thoracotomy cases had CRP peaks ~40–50% higher than VATS on average (Table 2), suggesting more surgical trauma leads to a greater inflammatory surge—a trend aligning with prior studies [44] that found significantly lower IL-6 and CRP levels with minimally invasive lung resections. Our inability to declare these differences significant likely reflects our sample size (see above) rather than a true absence of effect. Similarly, patients with infections showed a distinctly prolonged CRP elevation (often remaining > 100 mg/L at POD5) compared to uncomplicated patients, echoing findings from other surgical cohorts that persistent CRP indicates complication. However, we also observed overlap: some patients without infections had high CRP values postoperatively (e.g., a few open surgery patients without infection still had CRP ~120–150 mg/L on POD4). This reminds us that CRP is a non-specific marker—surgical inflammation alone can keep CRP elevated [45], or factors like preoperative inflammation and surgical extent can prolong CRP release. Thus, our study reinforces that while CRP trends are useful, they are not foolproof discriminators. We critically interpret our negative statistical results as likely due to type II errors or confounders rather than evidence that approach or extent has zero effect on CRP. Indeed, it would contradict immunological principles if a muscle-sparing VATS and a muscle-dividing thoracotomy induced identical inflammatory responses—instead, the issue is our data’s variability. We noted moderate effect sizes (e.g., Pillai’s Trace for time × N stage = 0.65) that did not reach significance, hinting that a larger study might find real differences where we could not. By integrating these points, we present a balanced view: CRP kinetics show expected patterns in our cohort, but confirming their statistical and clinical significance requires caution and further research.

In practical terms, our data support a management algorithm wherein a CRP that fails to fall (or rises anew) after POD3 should trigger a thorough evaluation for complications. Specifically, clinicians should consider obtaining imaging (a chest radiograph at minimum, and often a CT scan for more detailed inspection) to check for pneumonia, pleural collection (empyema), or anastomotic leak in esophageal or bronchial stumps. Simultaneously, appropriate microbiological cultures should be sent—for example, sputum culture if pneumonia is suspected, blood cultures if fever or systemic signs are present, and pleural fluid culture if an effusion is seen. The surgical incisions and any chest tube sites must be examined for signs of infection (erythema, drainage). In our practice, we involve the multidisciplinary team early. For instance, if CRP remains >100 mg/L on day 4–5 without an obvious cause, we confer with infectious disease specialists and often perform a bronchoscopy or ultrasound as needed to localize infection. Empiric broad-spectrum antibiotics may be initiated while awaiting results, especially if the patient has clinical signs of sepsis. On the other hand, if the CRP trend is reassuringly downward and the patient appears well, one might avoid unnecessary invasive tests or prolonged antibiotics. Thus, incorporating CRP trends into postoperative care can improve decision-making—a persistently elevated CRP warrants an aggressive search for a problem, whereas a declining CRP can reinforce confidence in an uncomplicated recovery.

Our cohort size (*n* = 144) is relatively small compared to other studies on postoperative CRP [18]. This limited sample reduces statistical power, increasing the risk of false-negative results (Type II error), and it may not capture the full variability seen in larger populations. Consequently, the strength of our conclusions is limited, and generalizability is cautious. For context, some thoracic surgical studies have analyzed CRP in cohorts of several hundred to over a thousand patients, far exceeding our sample. Our negative findings (lack of significant differences by approach, extent, or infection) should therefore be interpreted with care—they may reflect insufficient power rather than a true absence of effect.

Early postoperative CRP monitoring in thoracic surgery provides valuable insight into patient recovery. In our cohort, CRP followed a predictable trajectory—rising to a peak around 2–3 days after lung resection and then decreasing in uncomplicated cases. We did not find statistically significant differences in CRP trends based on surgical approach (VATS vs. open) or extent of resection, likely owing to variability and sample size, but numerically the inflammatory response was greater with more invasive surgery. Importantly, persistently elevated CRP beyond post-op day 3–4 was associated with the occurrence of infection and complications. While our study was underpowered to establish firm cutoff values, the absence of a CRP decline in the late postoperative period should raise suspicion for occult problems. In clinical practice, CRP is best used as a trending tool: a normalizing CRP trend can reassure the team, whereas a high or rising CRP trend should prompt diagnostic imaging, cultures, and possibly preemptive therapy. Prospective studies are warranted to confirm these findings and to refine CRP-based thresholds for early intervention in thoracic surgical patients.

A key limitation is the absence of systematically collected comorbidity data. Pro-inflammatory conditions (diabetes, autoimmune disease) may elevate baseline CRP and modulate early postoperative kinetics; without these variables, we could not adjust for potential confounding. This constraint may bias estimates toward the null and partly explain non-significant between-group differences. Future prospective studies should incorporate standardized comorbidity capture (Charlson index) to enable adjusted analyses. We did not undertake a post hoc “persistent-CRP” subgroup analysis because the retrospective design, time-dependent availability of POD3–4 CRP testing, and limited covariate capture would introduce selection bias and multiplicity; a prospective, pre-specified study is needed to derive and validate actionable thresholds.

## Figures and Tables

**Table 1:** Patient demographics and baseline characteristics.

Characteristic	Overall (*n* = 144)	VATS (*n* = 79)	Open Thoracotomy (*n* = 65)	*p*-Value
**Age, years (mean ± SD)**	61.3 ± 11.1	61.0 ± 10.4	61.6 ± 12.1	0.74
**Sex—Male**	90 (62.5%)	42 (53.2%)	48 (73.8%)	0.017
**Sex—Female**	54 (37.5%)	37 (46.8%)	17 (26.2%)	
**Mediastinoscopy—Yes**	41 (28.5%)	9 (11.4%)	32 (49.2%)	1.425 × 10^−6^
**Mediastinoscopy—No**	103 (71.5%)	70 (88.6%)	33 (50.8%)	
**Tumor side—Right**	84 (58.3%)	46 (58.2%)	38 (58.5%)	1
**Tumor side—Left**	57 (39.6%)	31 (39.2%)	26 (40.0%)	
**Extent—Segmentectomy**	25 (17.4%)	21 (26.6%)	4 (6.2%)	1.187 × 10^−5^
**Extent—Lobectomy**	96 (66.7%)	55 (69.6%)	41 (63.1%)	
**Extent—Bilobectomy**	9 (6.2%)	2 (2.5%)	7 (10.8%)	
**Extent—Pneumonectomy**	14 (9.7%)	1 (1.3%)	13 (20.0%)	

**Table 2:** Descriptive statistics of CRP (mg/L) at each measurement time point (P0–P5) by surgical approach (VATS vs. open thoracotomy) and extent of resection. Values are means and standard deviations (SD). *P0: preoperative baseline; P1–P5: postoperative days 1–5*.

Surgical Approach	Extent of Resection	CRP P0 (Mean ± SD)	CRP P1 (Mean ± SD)	CRP P2 (Mean ± SD)	CRP P3 (Mean ± SD)	CRP P4 (Mean ± SD)	CRP P5 (Mean ± SD)
**VATS**	Segmentectomy	80.45 ± 107.69	120.40 ± 95.88	178.35 ± 35.29	205.65 ± 112.78	120.95 ± 41.65	97.35 ± 61.45
	Lobectomy	3.25 ± 1.63	25.75 ± 17.32	132.10 ± 3.68	115.75 ± 11.81	120.65 ± 71.49	151.60 ± 146.94
	Pneumonectomy	8.20 ± –	92.10 ± –	140.70 ± –	156.40 ± –	119.70 ± –	165.70 ± –
	**Total VATS**	**35.12 ± 67.95**	**76.88 ± 68.45**	**152.32 ± 29.86**	**159.84 ± 72.38**	**120.58 ± 41.37**	**132.72 ± 86.12**
**Open Thoracotomy**	Segmentectomy	156.60 ± –	188.20 ± –	203.30 ± –	125.90 ± –	91.50 ± –	53.90 ± –
	Lobectomy	13.35 ± 13.52	56.41 ± 26.55	196.84 ± 85.34	192.91 ± 91.27	146.64 ± 94.73	103.06 ± 69.36
	Pneumonectomy	22.32 ± 15.06	78.60 ± 23.02	273.72 ± 46.34	271.38 ± 73.88	219.12 ± 94.25	156.46 ± 66.99
	Bilobectomy	4.05 ± 2.62	41.95 ± 29.63	155.65 ± 10.96	140.90 ± 13.15	78.25 ± 25.95	77.55 ± 30.90
	**Total Open**	**23.94 ± 37.85**	**69.77 ± 41.09**	**216.12 ± 76.04**	**206.74 ± 88.67**	**157.29 ± 95.17**	**113.49 ± 67.73**
**Combined Total**	**(All patients)**	**26.60 ± 44.96**	**71.47 ± 47.04**	**200.93 ± 72.73**	**195.58 ± 85.82**	**148.55 ± 85.98**	**118.07 ± 70.67**

**Table 3:** Multivariate test results for within-subject effects (Time) and interactions with between-subject factors on CRP levels (Pillai’s Trace criterion). Each row represents a multivariate test of the effect of time or a time × factor interaction.

Effect	Pillai’s Trace	F	Sig. (*p*)	Partial η^2^
**Time (within-subject)**	0.413	0.562	0.73	0.413
**Time × Surgical approach**	0.524	0.882	0.564	0.524
**Time × Extent of resection**	0.885	0.502	0.908	0.295
**Time × Age**	0.353	0.436	0.806	0.353
**Time × Gender**	0.484	0.749	0.627	0.484
**Time × Mediastinoscopy**	0.198	0.198	0.947	0.198
**Time × Tumor position**	0.08	0.069	0.994	0.08
**Time × Side**	0.148	0.139	0.974	0.148
**Time × Histology**	0.35	0.430	0.81	0.35
**Time × T stage**	0.551	0.982	0.521	0.551
**Time × N stage**	0.651	1.492	0.36	0.651

**Table 4:** Tests of within-subject effects (repeated-measures ANOVA) for the effect of time and time × group interactions on CRP levels. Degrees of freedom (df) and F-statistics are Greenhouse–Geisser corrected where applicable. *None of the effects reached statistical significance at α = 0.05*.

Source of Variation	Type III SS	df	Mean Square	F	Sig. (*p*)	Partial η^2^
**Time (overall effect)**	16,807.9	5 (GG *ε* = 2.251)	3361.581	1.127	0.352	0.124
**Time × Surgical approach**	15,537.11	5 (GG *ε* = 2.251)	3107.421	1.042	0.381	0.115
**Time × Extent of resection**	50,789.57	15 (GG *ε* = 6.753)	3385.971	1.136	0.384	0.299
**Time × Age**	10,231.5	5 (GG *ε* = 2.251)	2046.3	0.686	0.533	0.079
**Time × Gender**	19,933.53	5 (GG *ε* = 2.251)	3986.706	1.337	0.29	0.143
**Time × Mediastinoscopy**	5119.35	5 (GG *ε* = 2.251)	1023.87	0.343	0.738	0.041
**Time × Tumor position**	4202.609	5 (GG *ε* = 2.251)	840.522	0.282	0.782	0.034
**Time × Side**	3583.207	5 (GG *ε* = 2.251)	716.641	0.24	0.813	0.029
**Time × Histology**	2911.991	5 (GG *ε* = 2.251)	582.398	0.195	0.848	0.024
**Time × T stage**	10,254.75	5 (GG *ε* = 2.251)	2050.949	0.688	0.532	0.079
**Time × N stage**	4632.105	5 (GG *ε* = 2.251)	926.421	0.31	0.905	0.038

**Table 5:** Within-subject contrast results for specific time-interval comparisons of CRP, and interactions of these time-interval differences with surgical approach and extent of resection.

Within-Subject Contrast	df	F	Sig. (*p*)	Partial η^2^
**Time: P0 vs. P1**	1	0.246	0.633	0.03
Time: P1 vs. P2	1	2.319	0.166	0.225
Time: P2 vs. P3	1	0.123	0.735	0.015
Time: P3 vs. P4	1	1.482	0.258	0.156
Time: P4 vs. P5	1	0	0.996	0
**Time × Surgical approach: P0 vs. P1**	1	0.03	0.868	0.004
Time × Surgical approach: P1 vs. P2	1	1.532	0.251	0.161
Time × Surgical approach: P2 vs. P3	1	0.018	0.897	0.002
Time × Surgical approach: P3 vs. P4	1	0.248	0.632	0.03
Time × Surgical approach: P4 vs. P5	1	4.998	0.056	0.385
**Time × Extent: P0 vs. P1 (df = 3)**	3	0.431	0.737	0.139
Time × Extent: P1 vs. P2 (df = 3)	3	1.674	0.249	0.386
Time × Extent: P2 vs. P3 (df = 3)	3	0.543	0.666	0.169
Time × Extent: P3 vs. P4 (df = 3)	3	0.714	0.571	0.211
Time × Extent: P4 vs. P5 (df = 3)	3	0.625	0.619	0.19

## Data Availability

De-identified data and analysis code are available from the corresponding author upon reasonable request and subject to institutional policies.
